# Giant nonlinear Hall effect in twisted bilayer WSe_2_

**DOI:** 10.1093/nsr/nwac232

**Published:** 2022-10-22

**Authors:** Meizhen Huang, Zefei Wu, Jinxin Hu, Xiangbin Cai, En Li, Liheng An, Xuemeng Feng, Ziqing Ye, Nian Lin, Kam Tuen Law, Ning Wang

**Affiliations:** Department of Physics and Center for Quantum Materials, The Hong Kong University of Science and Technology, Hong Kong 999077, China; Department of Physics and Center for Quantum Materials, The Hong Kong University of Science and Technology, Hong Kong 999077, China; Department of Physics and Center for Quantum Materials, The Hong Kong University of Science and Technology, Hong Kong 999077, China; Department of Physics and Center for Quantum Materials, The Hong Kong University of Science and Technology, Hong Kong 999077, China; Department of Physics and Center for Quantum Materials, The Hong Kong University of Science and Technology, Hong Kong 999077, China; Department of Physics and Center for Quantum Materials, The Hong Kong University of Science and Technology, Hong Kong 999077, China; Department of Physics and Center for Quantum Materials, The Hong Kong University of Science and Technology, Hong Kong 999077, China; Department of Physics and Center for Quantum Materials, The Hong Kong University of Science and Technology, Hong Kong 999077, China; Department of Physics and Center for Quantum Materials, The Hong Kong University of Science and Technology, Hong Kong 999077, China; Department of Physics and Center for Quantum Materials, The Hong Kong University of Science and Technology, Hong Kong 999077, China; Department of Physics and Center for Quantum Materials, The Hong Kong University of Science and Technology, Hong Kong 999077, China; William Mong Institute of Nano Science and Technology, The Hong Kong University of Science and Technology, Hong Kong 999077, China

**Keywords:** nonlinear Hall effect, moiré superlattice, twisted bilayer WSe_2_, continuous Mott transition, electron-electron correlation effect

## Abstract

The recently discovered nonlinear Hall effect (NHE) in a few non-interacting systems provides a novel mechanism for generating second-harmonic electrical Hall signals under time-reversal-symmetric conditions. Here, we introduce a new approach to engineering an NHE by using twisted moiré structures. We found that the twisted WSe_2_ bilayer exhibited an NHE when the Fermi level was tuned to the moiré flat bands. When the first moiré band was half-filled, the nonlinear Hall signal exhibited a sharp peak with a generation efficiency that was at least two orders of magnitude greater than those obtained in previous experiments. We discuss the possible origins of the diverging generation efficiency in twisted WSe_2_ based on resistivity measurements, such as moiré-interface-induced correlation effects and mass-diverging-type continuous Mott transition. This study demonstrates not only how interaction effects can combine with Berry curvature dipoles to produce novel quantum phenomena, but also the potential of NHE measurements as a new tool for studying quantum criticality.

## Teaser

They report a giant nonlinear Hall generation efficiency of 1000 V^−1^ in small-angle-twisted bilayer WSe_2_, which is two orders of magnitude higher than previous records, and interpret the result by the correlation induced continuous Mott transition effect.

## INTRODUCTION

The nonlinear Hall effect (NHE) can arise intrinsically from the Berry curvature dipole (BCD) moments of two-dimensional (2D) materials [1]. Unlike linear Hall effects, the NHE possesses a transverse voltage oscillating at twice the frequency of the driving alternating current (AC) and a direct-current (DC) signal that is converted from the driving alternating current. Materials with a tunable nonlinear Hall response can be used in a broad range of technological applications that require second-harmonic generation or rectification, such as efficient energy harvesting, next-generation wireless techniques and infrared detectors [2–4]. The rectification and frequency doubling through the NHE are achieved by the intrinsic properties of the material, and therefore do not have the thermal voltage thresholds and/or the transition time characteristic of semiconductor junctions/diodes [4,5]. To date, however, experimental observations of the NHE have been largely limited to a small class of non-centrosymmetric materials with non-zero Berry curvatures (BCs), such as WTe_2_ [6,7], strained MoS_2_ [8] and corrugated graphene [9], in which the broken inversion and rotational symmetries allow finite BCDs to emerge [10,11].

The development of van der Waals assembling techniques offers exciting opportunities to engineer heterostructures with exotic physical properties beyond those of the individual materials [12,13]. For example, when stacking two monolayer WSe_2_ together with a small twist angle, the interlayer hybridization opens an energy bandgap at the band folding point and produces flat sub-bands [14,15]. The modified band structures have led to observation of emergent phenomena related to electron correlations beyond the expectations from single particle physics, such as continuous Mott transition and possible superconducting phases [16–19]. In this study, we demonstrate that the twisting technique can provide an additional route for engineering the NHE. We report that a giant NHE occurs near the electron-correlation-induced insulating states at half-filling of the twisted WSe_2_ (tWSe_2_) moiré bands. Near half-filling, we observe a giant second-harmonic Hall voltage $V_ \bot ^{2\omega }$ of ∼15 mV driven by a longitudinal voltage $V_{//}^\omega $ of ∼3.5 mV. The corresponding nonlinear Hall generation efficiency *η*, defined as $V_ \bot ^{2\omega }/{( {V_{//}^\omega } )}^2$, reaches 10^3^ V^−1^, which is at least two orders of magnitude higher than the maximum value in non-twisted materials reported thus far [9]. Although the standard theory proposed by Sodemann and Fu can be used to understand the experimental data away from half-filling [1], the giant enhancement of the nonlinear Hall signal cannot be understood even when the moiré bands and strain-induced BCDs are taken into account. We noticed a clue that could help explain the generation of giant NHE efficiency $\eta $, which is proportional to the effective mass of the quasiparticles. From the temperature (*T*) dependence of the device channel resistance, we found that there is a metal–insulator transition near half-filling showing similar features to the continuous Mott transition with mass-diverging characteristics, and the giant NHE occurs near this phase transition point. Although there is no theory available to link the giant NHE with the metal–insulator transition near half-filling, the demonstration of the strongly enhanced NHE efficiency $\eta $ provides a new experimental tool that can be used to understand the nature of the metal-to-Mott-insulator transition, which is one of the most challenging and interesting problems in condensed matter physics [20–23].

## RESULTS AND DISCUSSION

### Nonlinear Hall signals in tWSe_2_

**Figure 1.**
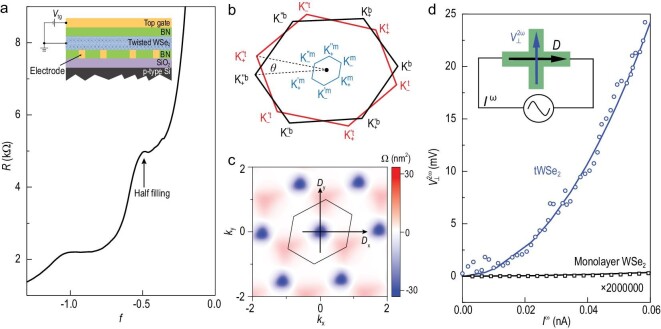
Strain-induced Berry curvature dipole in tWSe_2_. (a) *R* plotted as a function of *f* for a standard tWSe_2_ sample measured at *T* = 1.5 K. Inset: Schematic of the device structure. (b) First Brillouin zone of the top layer (outer black hexagon labeled with t), bottom layer (outer red hexagon labeled with b) WSe_2_ and the moiré Brillouin zone (inner blue hexagon) with strain. (c) BC of the top moiré valence band with a strain strength of 0.6% along the zigzag direction. The unbalanced BC distribution results in a finite dipole along both zigzag (*x*) and armchair (*y*) directions. (d) Blue circles: $V_ \bot ^{2\omega }$ as a function of ${I}^\omega $ measured at half-filling of tWSe_2_ at *T* = 1.5 K. Black squares: $V_ \bot ^{2\omega }$ measured in monolayer WSe_2_. The dots are experimental data and the solid lines are parabolic fits of the data. Inset: Illustration of the NHE. A driving alternating current parallel to the dipole (*D*) can generate a second-harmonic nonlinear Hall voltage.

Dual-gate devices composed of metal top gate/hexagonal boron nitride (hBN)/tWSe_2_/bottom contacts/hBN were fabricated, as schematically shown in the inset of Fig. 1a. We used platinum for the bottom electrodes to ensure good contact with the valence band of WSe_2_. Figure 1a shows the resistance *R* of a typical high-quality tWSe_2_ (twist angle $\theta \ = \ 2.0$°) as a function of the filling *f* (where *f* = −1 indicates that there are two holes per moiré unit cell) at the temperature *T* = 1.5 K. The appearance of a resistance peak at half-filling indicates the presence of strong correlation effects within the first moiré band, which is consistent with the results reported from other high-quality twisted moiré devices [12,13,16,17].

It is important to note that unstrained tWSe_2_ is not expected to exhibit the NHE because of its 3-fold rotational symmetry, which forces the BCD to be zero. However, based on our scanning transmission electron microscopy (STEM) and scanning tunnelling microscope (STM) characterization for tWSe_2_ samples with $\theta $ ranging from 1° to 4°, we found that strain-induced 3-fold rotational symmetry breaking (estimated to be in the range of 0.2%∼0.9%; see Supplementary Fig. 1) was universally present in the fabricated devices [24]. As illustrated in the calculation (shown in Fig. 1b and c), the strain reduces the symmetry of the tWSe_2_ from D_3_ to C_1_, changes the BC distribution, and induces non-zero BCD, allowing the NHE to occur (see Supplementary Fig. 2 for details).

To measure the NHE, we applied a driving alternating current ${I}^\omega $ with frequency $\omega $ between the source and the drain (shown in the inset of Fig. 1d), and measured the Hall voltage $V_ \bot ^{2\omega }$ oscillating at $2\omega $. At half-filling of a $\theta \ = \ 2.0$° sample, $V_ \bot ^{2\omega }$ increased nonlinearly and scaled quadratically with ${I}^\omega $ (plotted as blue circles; Fig. 1d), exhibiting a clear nonlinear charge response. For comparison, we also collected data from a monolayer WSe_2_ device with the same device structure (enlarged $2 \times {10}^6$ times and plotted as black squares; Fig. 1d). In contrast, $V_ \bot ^{2\omega }$ for monolayer WSe_2_ could hardly be observed at a similar carrier density of *p* ∼ 1 × 10^12^ cm^−2^. In analogy to the spin Hall angle, which measures the efficiency of the spin–charge conversion, the nonlinear Hall generation efficiency can be expressed as *η* = $V_ \bot ^{2\omega }/{( {V_{//}^\omega } )}^2$ (a comparison between the generation efficiency, responsivity and nonlinear conductivity can be found in Supplementary Note. 1). For tWSe_2_, the observed nonlinear Hall generation efficiency *η* ∼10^3^ V^−1^ is orders of magnitude higher than the values for the monolayer WSe_2_ sample (*η* ∼ 0.017 V^−1^) and other non-interacting systems (see Supplementary Table 1 for a comparison) [6,7,9,25–27].

### Giant NHE at half-filling

**Figure 2.**
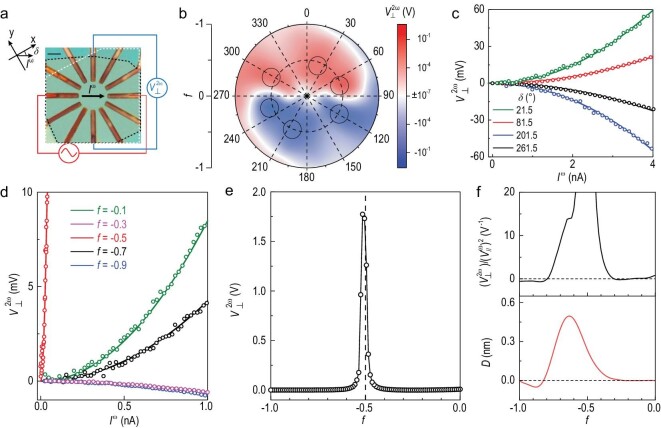
Giant nonlinear Hall effect in tWSe_2_. (a) Optical image of a disc-shaped sample (scale bar 4 ${\rm{\mu }}$m). The black (white) dashed area corresponds to the bottom (top) WSe_2_ layer. *x* is the zigzag direction and *y* is the armchair direction of the WSe_2_ crystal. ${I}^\omega $ is injected at angle $\delta $ from the zigzag direction. (b) Angular- and filling-dependent $V_ \bot ^{2\omega }$ measured at ${I}^\omega = {\rm{\ }}0.5{\rm{\ nA}}$ at *T* = 1.5 K. (c) $V_ \bot ^{2\omega }$ versus ${I}^\omega $ measured along different directions at *f* = −0.74 at *T* = 1.5 K. (d) $V_ \bot ^{2\omega }$ versus ${I}^\omega $ measured at different fillings along $\delta \ = \ 81.5^\circ $. The red curve at *f* = −0.5 is the same one as the blue curve shown in Fig. 1d. The dots are experimental data and the solid lines are parabolic fits of the data. (e) $V_ \bot ^{2\omega }$ along $\delta \ = \ 81.5^\circ $. A sharp peak is observed near the half-filling. (f) $V_ \bot ^{2\omega }/{( {V_{//}^\omega } )}^2$ extracted from the experiment (upper panel) compared with *D* calculated from theory (bottom panel) along $\delta \ = \ 81.5^\circ $.

As the nonlinear Hall signal strength reached maximum when the applied current was parallel to the dipole [1], we used a disc-shaped device to study the angular- and filling-dependence of the NHE (see Supplementary Fig. 3 for details). Figure 2a shows the optical image of the disc-shaped sample. We applied a driving alternating current ${I}^\omega $ between a pair of the 12 electrodes at an angle $\delta $ from the zigzag direction of the WSe_2_ crystal, and measured the second-harmonic voltage $V_ \bot ^{2\omega }$. The polar contour plot of $V_ \bot ^{2\omega }$ is shown in Fig. 2b, where the angular axis denotes the direction of current injection and the radial axis denotes the filling. The angular- and filling-dependent $V_ \bot ^{2\omega }$ exhibits two features. First, $V_ \bot ^{2\omega }$ switches signs when the current direction and the voltage probe connection are reversed simultaneously, indicating a BCD-induced nonlinear charge response (this can also be seen in Fig. 2c). Second, near the half-filling of the moiré band, $V_ \bot ^{2\omega }$ is orders of magnitude higher than the other fillings (represented by the black circles in Fig. 2b; this can also be seen in Fig. 2e).

To be more specific, $V_ \bot ^{2\omega }$ was plotted versus ${I}^\omega $ at different fillings and versus *f* along $\delta \ = \ 81.5^\circ $ in Fig. 2d and e, respectively. As can be seen in Fig. 2d, the $V_ \bot ^{2\omega } - {I}^\omega $ characteristic is robust at all fillings. $V_ \bot ^{2\omega }$ increases nonlinearly and scales quadratically with ${I}^\omega $, exhibiting a clear NHE. While the quadratic $V_ \bot ^{2\omega } - {I}^\omega $ characteristic is robust at all fillings, the $V_ \bot ^{2\omega }$ signal depends strongly on the filling. As shown in Fig. 2e, $V_ \bot ^{2\omega }$ exhibits a sharp peak at half-filling that drops by three orders of magnitude slightly away from it. Although a sharp peak at half-filling is present along most directions of the current injection, and sample inhomogeneity may play a role in a few directions, the unusual sensitivity of the $V_ \bot ^{2\omega }$ signal to filling suggests that the strong correlation effects at half-filling may play an important role in generating the giant nonlinear response.

To understand the origin of the giant NHE, we note that besides the sharp peak near half-filling, a small peak is exhibited by the nonlinear Hall signal when the filling *f* is near −0.63, as shown in the upper panel of Fig. 2f. In the non-interacting case and small twist angle of *θ* = 2.0°, the band structure under strain and the BCD of the top moiré bands can be rather accurately described by the continuum model (see Supplementary Note 2 for details). The lower panel of Fig. 2f shows that the calculated BCD, *D*, exhibits a peak at *f = −*0.63, which is consistent with the experimental measurement (see Supplementary Fig. 4 for details). The model also captures the sign change of the nonlinear Hall signal near *f = −*0.30/*−*0.77. When the filling *f* is near 0, the BCD is almost zero, as observed in the experiment. However, the non-interacting electron model cannot explain the giant NHE near *f* = *−*0.5. As the large enhancement of the nonlinear Hall signals occurs near half-filling only, the natural deduction is that the interaction effects at half-filling should play a critical role in the NHE signal enhancement. The unique features of the metal–insulator transition near half-filling with mass-diverging characteristics could provide more insight into the causes of the giant NHE.

### Effective mass divergence and metal–insulator transition near half-filling states

For *θ* = 2.0°, the on-site Coulomb repulsion energy *U* of each site is estimated to be ∼47.6 meV (see Methods for calculation details). Our calculations show that the band width *W*_b_ of the first hole moiré band for *θ* = 2.0° is ∼10 meV (Supplementary Fig. 1). The large value of the ratio *U*/*W*_b_ suggests the possibility of a metal–insulator transition near the half-filling of the band [28]. Mott proposed a carrier–density–vanishing–type first-order metal–insulator transition mechanism [29]. In contrast, recent experiments on hetero-bilayer MoTe_2_/WSe_2_ and tWSe_2_ support the presence of a second-order metal–insulator transition [18,19].

**Figure 3.**
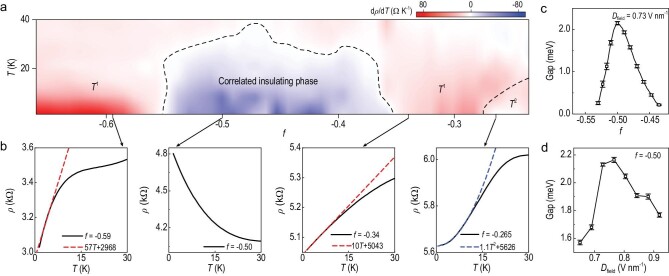
Mass-diverging-type continuous metal–insulator transition. (a) First derivative in temperature of the resistivity. (b) Plots of resistivity (black solid lines) versus *T* as well as linear (red) and quadratic (blue) fits (dashed lines) at different filling. (c) Plot of the measured insulating gap versus filling. (d) Plot of the measured insulating gap versus displacement field.

We carried out a detailed study on the correlated insulating and metallic phases in our tWSe_2_ samples by measuring the temperature-dependence of the resistance as shown in Fig. 3a and b. In the region −0.28 < *f* < −0.25, where *f* denotes the filling, the temperature-dependent resistivity up to 15 K can be expressed by the relation *ρ* = *ρ*_0_ + *αT*^2^ (where *ρ*_0_ is the residue resistivity and *α*^1/2^ is the fitting parameter proportional to the effective mass m*), which is Fermi liquid behaviour. The parameter *α*^1/2^ is enhanced as *f* approaches quantum criticality from the metallic side (see Supplementary Fig. 5). In contrast, for a range of fillings near the correlated insulator, *T*-linear behaviour is observed down to the lowest temperatures. Figure 3b shows the *R*-*T* curves near the metal–insulator transition point. The resistivity exhibits *T*-linear behaviour (*ρ* = *ρ*_0_ + *βT*) for −0.35 < *f* < −0.28 at temperature *T* < 10 K and saturates at high temperatures (see Supplementary Table 2 for the values of the fitting parameters α and *β*, which are similar to those in previous reports). Upon increasing the doping, a correlated insulating phase with ${\rm{d}}\rho /{\rm{d}}T < 0$ is observed. Further increasing the doping, *T*-linear behaviour is recovered down to the lowest temperatures. This ‘anomalous metallic’ behaviour is similar to that in some strongly correlated heavy-fermion metals [20], magic-angle twisted bilayer graphene [30] and, as described in recent works, in twisted transition metal dichalcogenides [18,19]. As shown in Fig. 3c, the extracted insulating gap as a function of the filling drops smoothly from the highest value at half-filling to nearly zero at the quantum critical point, indicating a continuous phase transition. Similar behaviour is also observed when tuning the displacement field (Fig. 3d), in which the size of the gap can be tuned by 30%, mainly due to the limited tuning capability of the gate voltages (see Supplementary Fig. 6 for the displacement electric field's effect).

**Figure 4.**
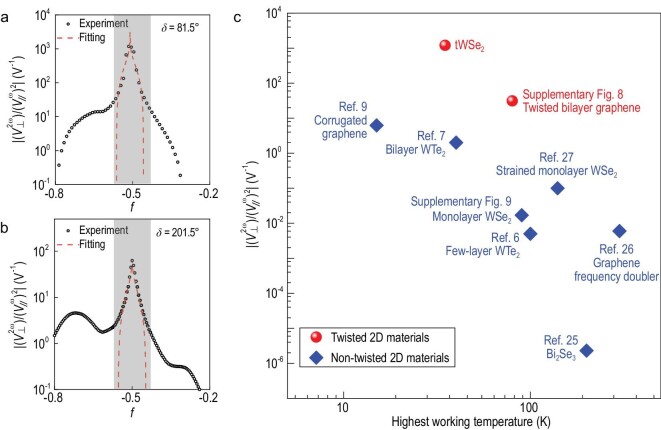
Giant nonlinear Hall response near the continuous Mott transition. (a, b) Black dots: $V_ \bot ^{2\omega }/{( {V_{//}^\omega } )}^2$ obtained along (a) $\delta \ = \ 81.5^\circ $ and (b) $\delta \ = \ 201.5^\circ $ from the experiment near the half-filling. Red dashed line: theoretical fitting using the effective mass divergence formula. The grey shaded area corresponds to the correlated insulating phase. (c) Logarithmic plot of $V_ \bot ^{2\omega }/{( {V_{//}^\omega } )}^2$ versus the highest working temperature for time-reversal-symmetric frequency doublers in various kinds of 2D materials.

It is important to note that the moiré lattice of the twisted bilayer WSe_2_ sample is triangular, and such a system is supposed to be described by a Hubbard model with triangular lattices. It has been predicted that such a Hubbard model supports a continuous metal-to-Mott-insulator transition [31–33]. Near the transition, the quasiparticle effective mass diverges [21]. Although the properties of the quasiparticles are greatly modified, we expect that their transport properties can still be described by the Boltzmann equation. As a result, the nonlinear Hall signal, which is proportional to both the effective mass and the BCD ($V_ \bot ^{2\omega } \propto D{m}^*$), and the nonlinear Hall generation efficiency (*η* = $V_ \bot ^{2\omega }/{( {V_{//}^\omega } )}^2$) also diverge as the effective mass diverges (see Supplementary Fig. 4 for details). As shown in Fig. 4a and b, $\eta $ extracted experimentally near half-filling can be well fitted by using ${m}^*/{m}_{e\ } \propto \ln \frac{1}{{| {1 - 2f} |}}$, where ${m}_{e\ }$ is the bare electron mass [21]. Besides, the width of the signal peak is found to be consistent with the width of the insulating region (see Supplementary Fig. 7).

### Giant NHE origins

The standard NHE theory proposed by Sodemann and Fu, which deals with the materials in the single-particle picture, is applicable when interpreting our experimental data away from half-filling [1]. Qualitatively, the nonlinear Hall signal is proportional to both *D* and the effective mass ${m}^*$: $V_ \bot ^{2\omega } \propto D{m}^*$. It is thus understandable that increasing the effective mass, when approaching the continuous metal–insulator transition, can result in the enhancement of the nonlinear Hall signal. However, around half-filling, the effective mass diverges. It is difficult to estimate the value of the enhanced effective mass since there is no theory available to carry out such a qualitative calculation. On the other hand, because of the insulating behaviour near half-filling, the effective mass cannot be easily measured by transport experiments. Although we are not able to provide detailed mechanisms to theoretically link the giant NHE signal to the metal–insulator transition or the half-filling states at this stage, our experimental data demonstrate that the giant NHE occurring at the metal–insulator transition regime near half-filling does not seem like a coincidence.

In addition to the effective mass divergence effect, there are other factors that need more attention. For example, the Fermi sea contribution, which is neglected in the general model [1], could generate a relatively high nonlinear Hall signal in the correlated insulator region owing to the small scattering time [34]. As a matter of fact, there is no theory applicable to quantitative estimation of the NHE under the situation of strong electron-electron correlation. The correlation effects could serve as a new mechanism for second-harmonic generation. To this end, new theories other than the BCD [35] and further experimental investigations are needed.

The twisted WSe_2_ bilayers provide a substantial platform for generating giant nonlinear Hall signals, which is reflected by their nonlinear Hall generation efficiency as compared to other kinds of 2D materials in Fig. 4c (also see Supplementary Figs 8–9 and refs. [6,7,9,25–27]). Among them, tWSe_2_ has the highest nonlinear Hall generation efficiency, which is 2–3 orders of magnitude higher than those of non-interacting systems. Twisted systems can generate a large nonlinear Hall signal with a tunable amplitude, making them an important addition to the arsenal of van der Waals technologies. They can be used, for example, in efficient energy harvesting, next-generation wireless techniques, infrared detectors and other applications that need second-harmonic generation or rectification. Our study can also help develop an additional route for generating and engineering BCDs that can also be applied to other 2D materials with non-zero BC.

## CONCLUSIONS

We demonstrate that tWSe_2_ is a highly tunable and correlated system for introducing and manipulating NHE. The giant NHE occurs at the critical regime of the metal-to-insulator transition near half-filling states of the moiré interface structure. Measurement of the diverging behaviour of the nonlinear Hall generation efficiency provides a new tool for studying the novel properties of moiré electronic systems and their correlation effects near the critical regime.

## METHODS

### Device fabrication

The transport devices in this study were fabricated using the tear and stack method. The hBN (∼30 nm) and WSe_2_ flakes are exfoliated on SiO_2_/Si substrates and identified optically. Pt electrodes (10 nm) are patterned on the bottom hBN. The top hBN (∼15–30 nm) is picked up from the SiO_2_/Si substrate using a polycarbonate/polydimethylsiloxane (PC/PDMS) stamp on a glass slide. We manually rotate the transfer stage by a small angle (1∼4°) between picking up two WSe_2_ pieces [36]. The entire top hBN/tWSe_2_ stack is transferred onto the pre-patterned Pt electrodes placed on the bottom hBN. After that, a Ti/Au (10/30 nm) top gate is patterned. The exfoliation and transfer of monolayer WSe_2_ flakes were performed in a glovebox equipped with a dry transfer set-up. Step-by-step fabrication details are illustrated in Supplementary Note 3. Our high-quality tWSe_2_ devices with superconductivity signatures were prepared using the same process.

### Transport measurement

Transport measurements were performed in a cryogenic system, which provides stable temperatures ranging from 1.4 to ∼300 K and fields up to 14 Tesla. The DC top gate was applied through our home-built DC power supply with a resolution of ∼1 mV. AC bias voltage was applied to the source probe through Stanford Research Systems DS360. The first- and second-harmonic signals were measured by Signal Recovery 7280 lock-in amplifier (impedance 100 MΩ).

### Carrier density, displacement field and twist angle determination

The carrier density *p* can be determined by a combination of the Hall measurement (see Supplementary Fig. 10) and carrier density induced by the top-gate voltage *V*_tg_ and the bottom-gate voltage *V*_bg_ based on a capacitor model: $pe\ = {C}_{\rm tg}\ ( {{V}_{{\rm{tg}}} - {V}_0} ) + {C}_{\rm bg}{V}_{{\rm{bg}}}$, where ${C}_{\rm tg}$ and ${C}_{\rm bg}$ are the capacitance of the top BN and the bottom SiO_2_ and ${V}_0$ is the threshold voltage at which the Fermi level touches the flat band edge. The displacement field can be calculated by ${D}_{{\rm{field}}} = ( {{C}_{\rm bg}{V}_{{\rm{bg}}} - {C}_{\rm tg}{V}_{{\rm{tg}}}} )/2{\varepsilon }_0\ $, where ${\varepsilon }_0$ denotes the vacuum dielectric constant. The twist angle is estimated from ${p}_0 = 2/( {\frac{{\sqrt 3 }}{2}{\lambda }^2} )\ $and $\lambda \ = a/( {2\sin \frac{\theta }{2}} )\ $where ${p}_0$ is the carrier density at the full-filling, and $a\ = \ 0.329\ {\rm{nm}}$ is the lattice constant of WSe_2_.

### Calculation of the on-site Coulomb energy

The on-site Coulomb energy *U* of each site is estimated to be ${e}^2/( {4\pi \varepsilon {d}_0} )$, where *e* is the elementary charge, $\varepsilon $ is the effective in-plane dielectric constant including screening and ${d}_0$ is the effective distance between each site [37]. Considering ${d}_0$ is 40% of the moiré wavelength and $\varepsilon = \,8{\varepsilon }_0$, we get $U\, = \,{e}^2\theta /( {4\pi {\varepsilon }_0a\kappa } )$, where $\kappa $ = 0.4$\ \times {\rm{\ }}$8 = 3.2 and *a* = 0.33 nm is the WSe_2_ lattice constant.

## Supplementary Material

nwac232_Supplemental_File
