# Supplementary material for: Giant nonlinear Hall effect in twisted bilayer WSe2
Source: Natl Sci Rev. 2022 Oct 22;10(4):nwac232. doi: 10.1093/nsr/nwac232 (PMC10171643; doi:10.1093/nsr/nwac232)
Supplement: nwac232_Supplemental_File [file nwac232_supplemental_file.docx]

Supplementary Information

for

**Giant nonlinear Hall effect in twisted bilayer WSe_2_**

Meizhen Huang^1, †^, Zefei Wu^1, †,^ *, Jinxin Hu^1, †^, Xiangbin Cai^1^, En Li^1^, Liheng An^1^, Xuemeng Feng^1^, Ziqing Ye^1^, Nian Lin^1^, Kam Tuen Law^1,^ *, Ning Wang^1,2,^ *

^1^Department of Physics and Center for Quantum Materials, The Hong Kong University of Science and Technology, Hong Kong, China

^2^William Mong Institute of Nano Science and Technology, The Hong Kong University of Science and Technology, Hong Kong, China

^†^These authors contributed equally: Meizhen Huang, Zefei Wu, Jinxin Hu.

*e-mail: phwang@ust.hk; phlaw@ust.hk; wzefei@connect.ust.hk


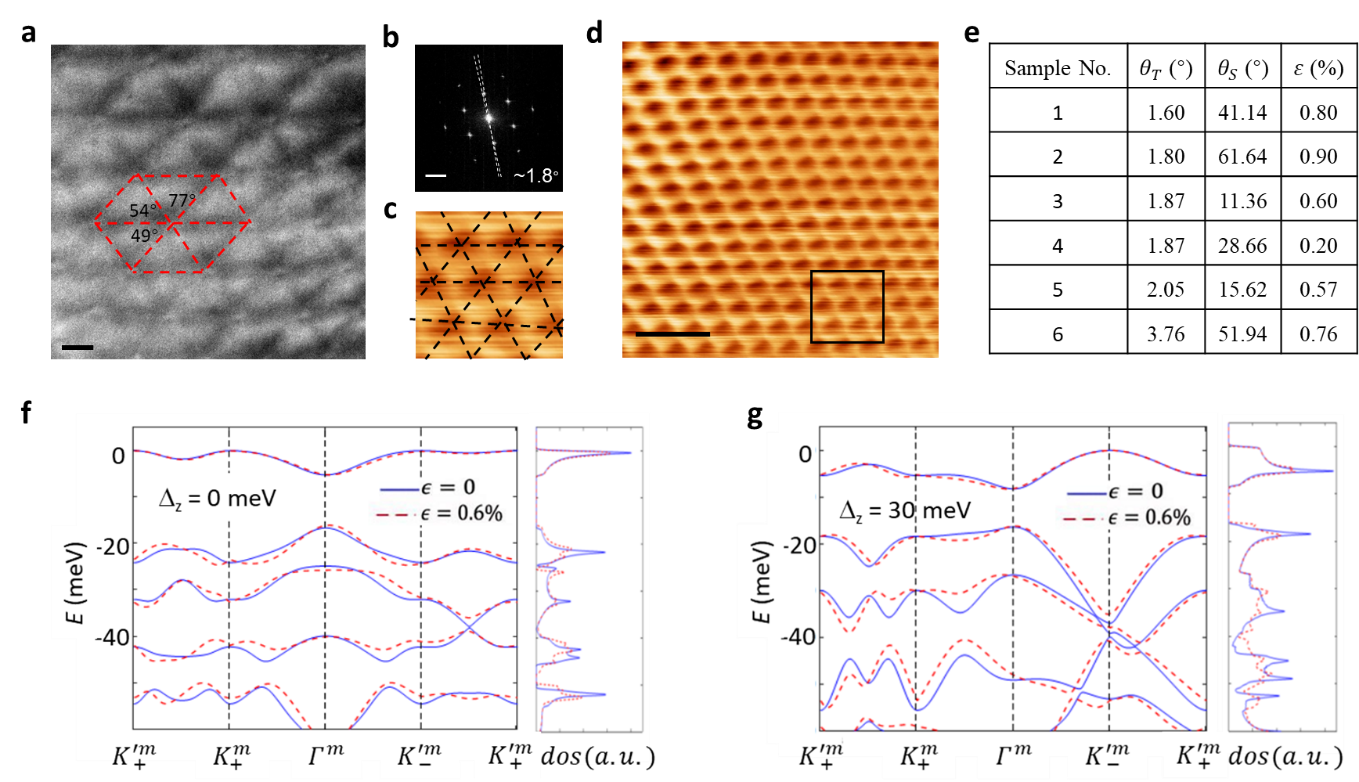


**Supplementary Fig. 1 | Symmetry breaking effects in tWSe_2_ sample. a,** High-resolution STEM image of the moiré superlattice formed from tWSe_2_ (scale bar 5 nm). AA stacking points (W atom on W atom or Se atom on Se atom) are connected through red lines. The moiré superlattice exhibits strong distortion as outlined by the red dashed lines connecting the nearest AA stacking points with angles of 77°, 54° and 49°, largely deviating from the ideal angle of 60°. Using the uniaxial heterostrain model^1^, the strain strength is calculated to be 0.90%. **b,** Twist angle (~1.8°) for the STEM sample can be determined by measuring the moiré lattice scale and the angular difference between the two sets of electron diffraction patterns. (scale bar 2.3 nm^-1^). **c,** Enlarged image of the region marked by the dark square in (**d**) (area length 19.2 nm). **d,** STM image from tWSe_2_ sample (scale bar 20 nm). The moiré superlattice is highly distorted and shows inhomogeneous strain strength. The strain strength is calculated to be 0.57% in this sample with a twist angle of 2.05°. e**,** Strain calculated in different STEM and STM samples. We conclude that significant symmetry breaking effects universally exist in tWSe_2_. **f,g,** Band structure and density of states (DOS) calculated at staggered layer potential $\Delta_{z}$ = 0 meV (**f**) and $\Delta_{z}$ = 30 meV (**g**), the later potential corresponds to the potential at half-filling (*f* = −0.5) in Fig. 2e.


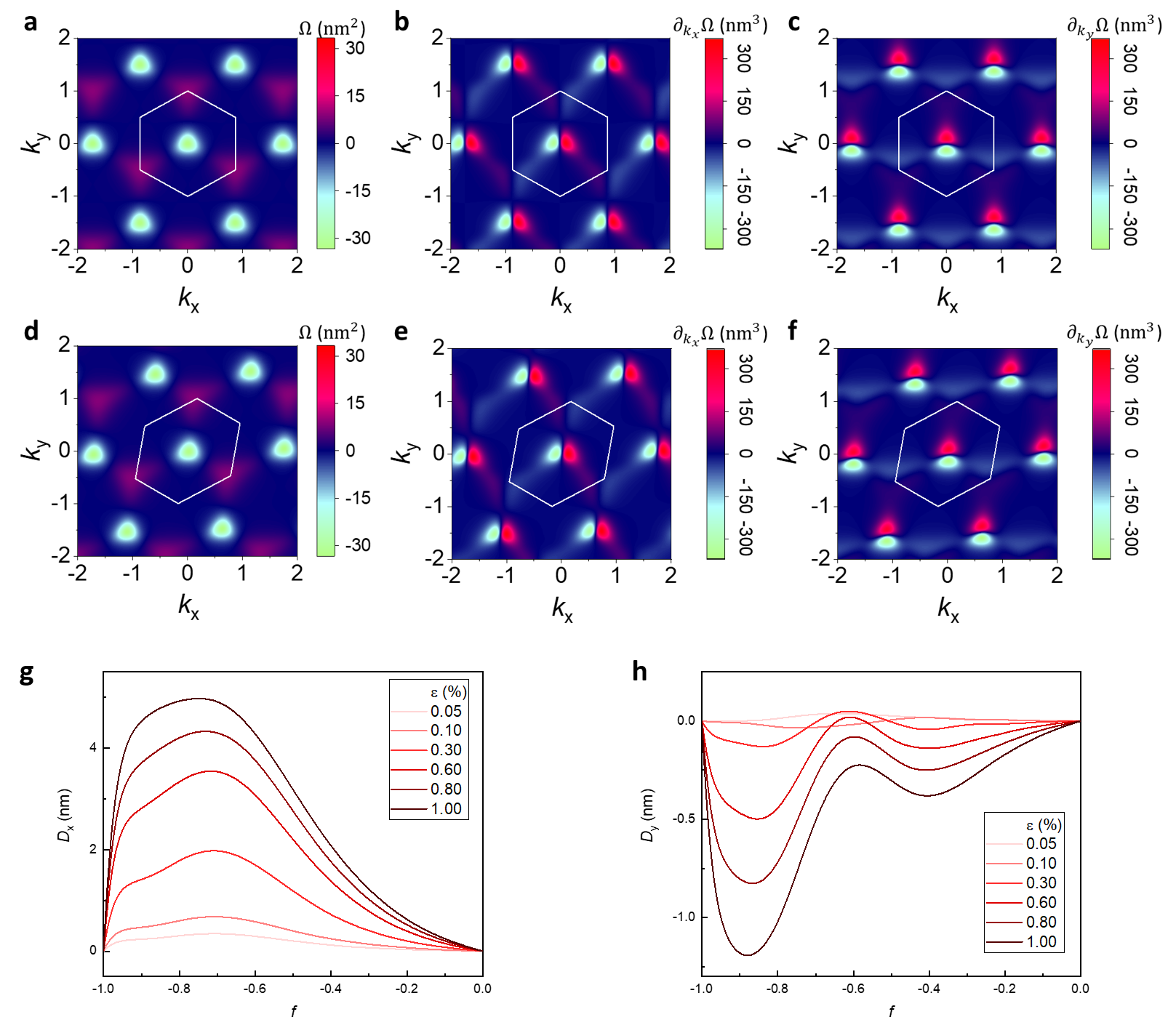


**Supplementary Fig. 2 | BC and BCD in tWSe_2_.** **a,** The Berry curvature of the top moiré valence band without strain. **b**-**c**, Dipole density along zigzag $k_{x}$ (**b**) and armchair $k_{y}$ (**c**) direction without strain. The BCD, which measures the gain in total BC flux, vanishes in this twisted system with C_3_ symmetry. $k_{x}$ and $k_{y}$ are in units of nm^-1^. **d**-**f,** The Berry curvature (**d**), dipole density along $k_{x}$ (**e**) and $k_{y}$ (**f**) direction after introducing a strain strength of 0.6% along the zigzag direction. The unbalanced BCD distribution is displayed in (**e**)/(**f**) along $k_{x}$/$k_{y}$ direction, respectively. This breaking of three-fold rotational symmetry results in finite BCD. The staggered layer potential is 30 meV in the calculation, which corresponds to half-filling (*f* = −0.5) in Fig. 2e. **g,h,** Theoretical values of *D_x_* (**g**) and *D_y_* (**h**) plotted as a function of filling based on a non-interacting model. *D_x_*(*D_y_*) is the BCD along the zigzag(armchair) direction. The giant enhancement of the nonlinear Hall signal at half-filling is irrelevant to the strain strength since *D* shows the same shape with a smooth amplitude change for different strain strengths.


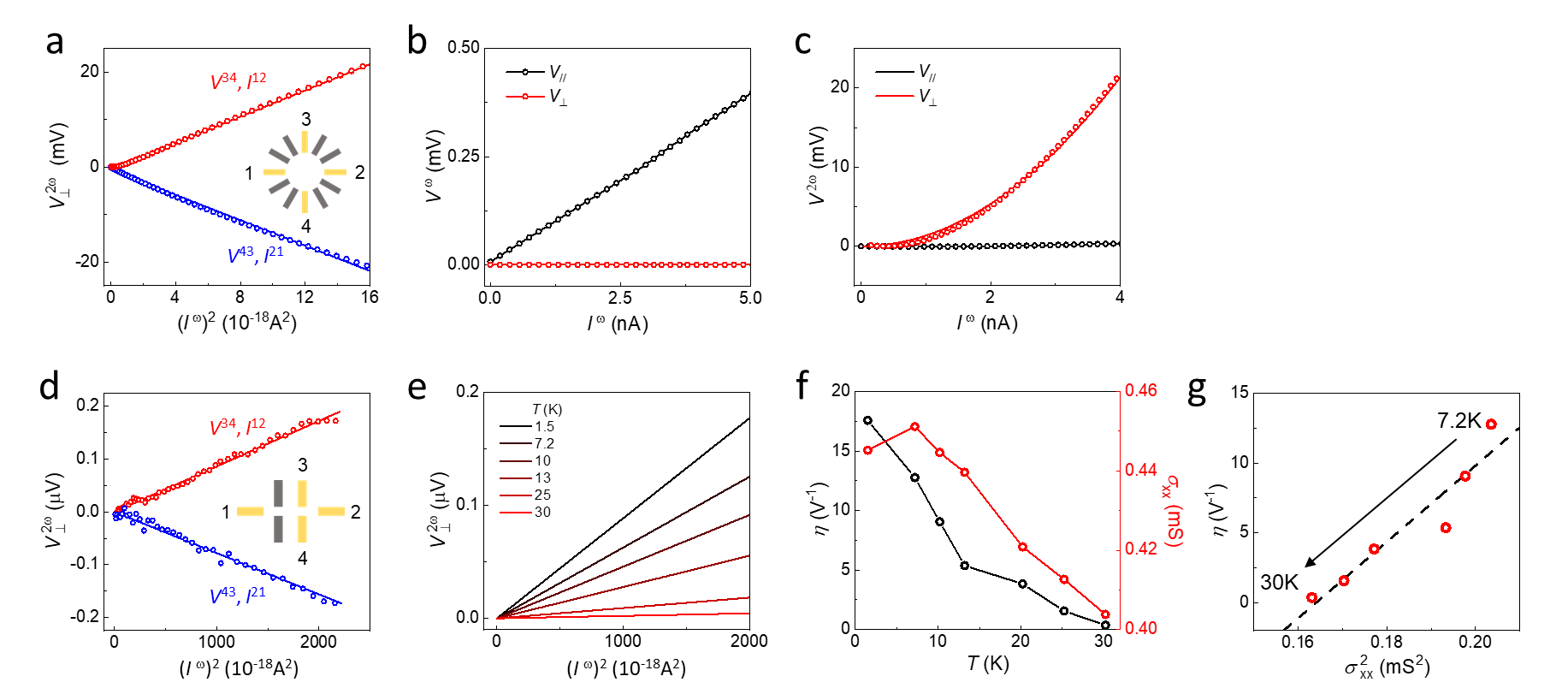


**Supplementary Fig. 3 | Additional NHE data of tWSe_2_. a,** $V_{\perp}^{2w}$ measured at *T* = 1.5 K at *f* = −0.74 from different combinations of electrodes as functions of $\left( I^{\omega} \right)^{2}$. Dots are measured values and lines are fitted curves. *V*^43^ and *I*^12^ indicate that the second harmonic voltage is measured through probes 4 and 3 when current $I^{\omega}$ is injected through probes 1 and 2. $V_{\perp}^{2\omega}$ scales quadratically with $I^{\omega}$ and changes its signs when both the voltage detection and current injection directions are reversed. Inset: electrode configuration of the device. **b,** The first harmonic longitudinal voltage $V_{⫽}^{\omega}$ measured at *T* = 1.5 K at *f* = −0.74 increases linearly with current $I^{\omega}$. By correcting the misalignment of the electrodes, nearly zero $V_{\perp}^{\omega}$ is obtained, suggesting that the device is a non-magnetic system with protected time-reversal symmetry. **c,** A second harmonic Hall response $V_{\perp}^{2\omega}$ measured at *T* = 1.5 K at *f* = −0.74 dominates over the longitudinal response $V_{⫽}^{2\omega}$. Dots are measured values and lines are parabolic fits of the data. All the features above verify the BCD-induced NHE in tWSe_2_ and exclude other possible effects such as the asymmetric flake shape, side-jump and skew-scattering, contact junction, and thermoelectric effects^2^.

(**d**)-(**g**) are the NHE data from another tWSe_2_ sample ($\theta=2.0$°). **d,** $V_{\perp}^{2w}$ measured at *T* = 1.5 K from different combinations of electrodes as functions of $\left( I^{\omega} \right)^{2}$. Inset: electrode configuration of the device. **e**, $V_{\perp}^{2\omega}$ measured at different temperatures as a function of ${(I^{\omega})}^{2}$. **f,** $\eta$ (left, black) and $\sigma_{xx}$ (right, red) as a function of *T*. **g,** $\eta$ as a function of $\left( \sigma_{xx} \right)^{2}$ at different temperatures. The dashed line is a linear fitting of experimental data. Though the linear fitting cannot rule out the possibility of other second harmonic transport mechanisms^3^, the conclusion that the BCD dominates in our samples is solid based on the Hall dominated second harmonic response.

**
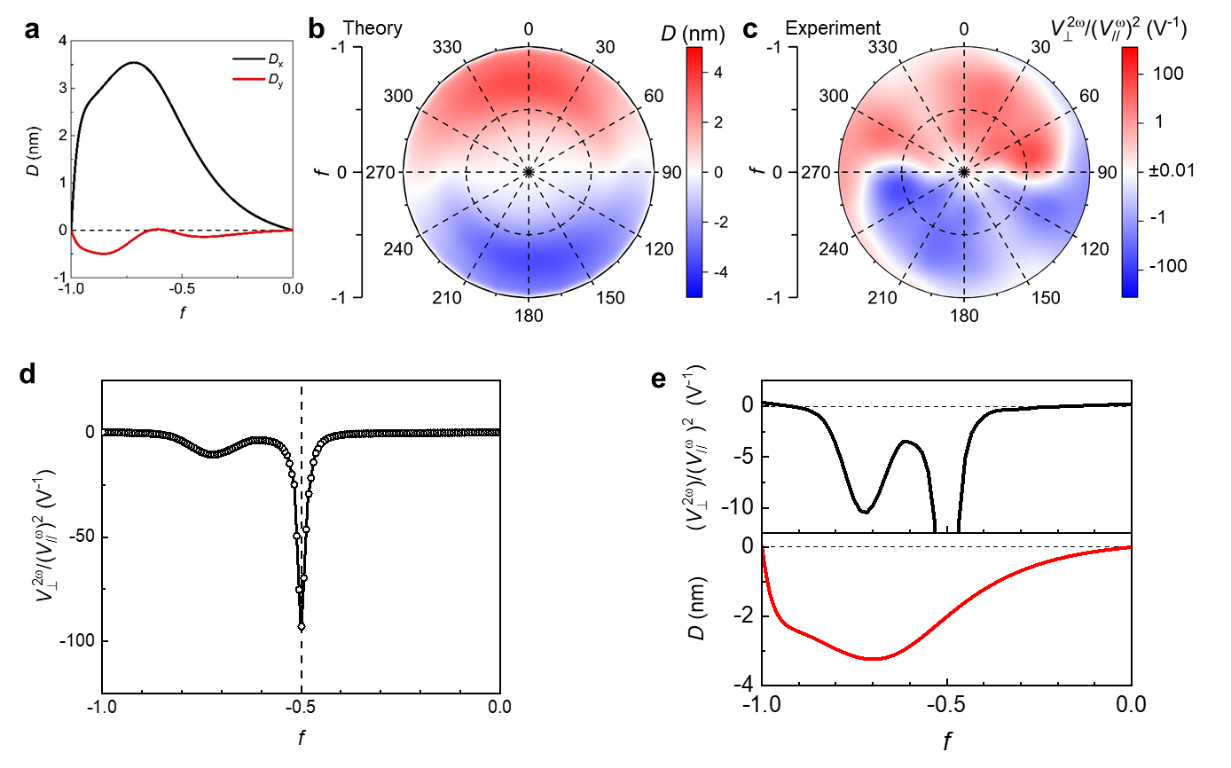
**

**Supplementary Fig. 4 | Theoretical calculation of the BCD and the NHE data at** $\boldsymbol{\delta=201.5^{\circ}}$**. a,** Calculated BCD along both zigzag ($D_{x}$) and armchair ($D_{y}$) directions using a continuum model. **b,** Angular and filling dependences of theoretical calculated ${D=D}_{x}cos\delta+D_{y}\sin\delta$ if we assume that the sample resistance is isotropic^4^. **c,** Angular and filling dependent ${V_{\perp}^{2\omega}}/{\left( V_{⫽}^{\omega} \right)^{2}}$ measured at *T* = 1.5 K. **d,** ${V_{\perp}^{2\omega}}/{\left( V_{⫽}^{\omega} \right)^{2}}$ extracted from experimental data along $\delta=201.5^{\circ}$. A sharp peak is observed near the half-filling. **e,** ${V_{\perp}^{2\omega}}/{\left( V_{⫽}^{\omega} \right)^{2}}$ extracted from experiment (upper panel) compared with *D* calculated from theory (bottom panel) along $\delta=201.5^{\circ}$. A small peak appears near filling ~ −0.73, which is consistent with the theoretical calculations.

The BCD for tWSe_2_ bilayers has both $D_{x}$ and $D_{y}$ components because the only symmetry is $C_{1}$. From Ref. 4, if we apply a voltage $V_{⫽}^{\omega}$ in the longitudinal direction, and measure the voltage $V_{\perp}^{2\omega}$ in the transverse direction, we can have:

$$\begin{aligned} \frac{V_{\perp}^{2\omega}}{\left( V_{⫽}^{\omega} \right)^{2}}=\frac{e^{3}\tau}{2\hbar^{2}}\frac{\rho_{xx}\rho_{yy}(D_{x}\rho_{xx}cos\delta+D_{y}\rho_{yy}\sin\delta)}{\left( \rho_{xx}\cos^{2} \delta+\rho_{yy}\sin^{2} \delta\right)^{2}} \end{aligned}$$

With $\delta$ is the angle between the driving current and $x$ direction, $\tau=\frac{m^{*}}{ne^{2}\rho}$ is the scattering time obtained from the Drude formula, $\rho_{xx}$ and $\rho_{yy}$ is the resistance along zigzag and armchair direction respectively. In our disc-shaped sample, if we assume that the resistance is isotropic ($\rho_{xx}=\rho_{yy}=\rho_{0}$), we have

$$\begin{aligned} \frac{V_{\perp}^{2\omega}}{\left( V_{⫽}^{\omega} \right)^{2}}=\frac{em^{*}}{2\hbar^{2}p}\left( D_{x}cos\delta+D_{y}\sin\delta\right)\propto Dm^{*}. \end{aligned}$$

Effectively, the total Berry curvature dipole ${D=D}_{x}cos\delta+D_{y}\sin\delta$.

**Supplementary Fig. 5 | Filling dependence of *α*^1/2^ in a log-log plot.** The parameter *α*^1/2^, which proportional to the quasiparticle effective mass $m^{*}$ according to Kadowaki–Woods scaling^5^, is enhanced as *f* is approached from the metallic side.

**
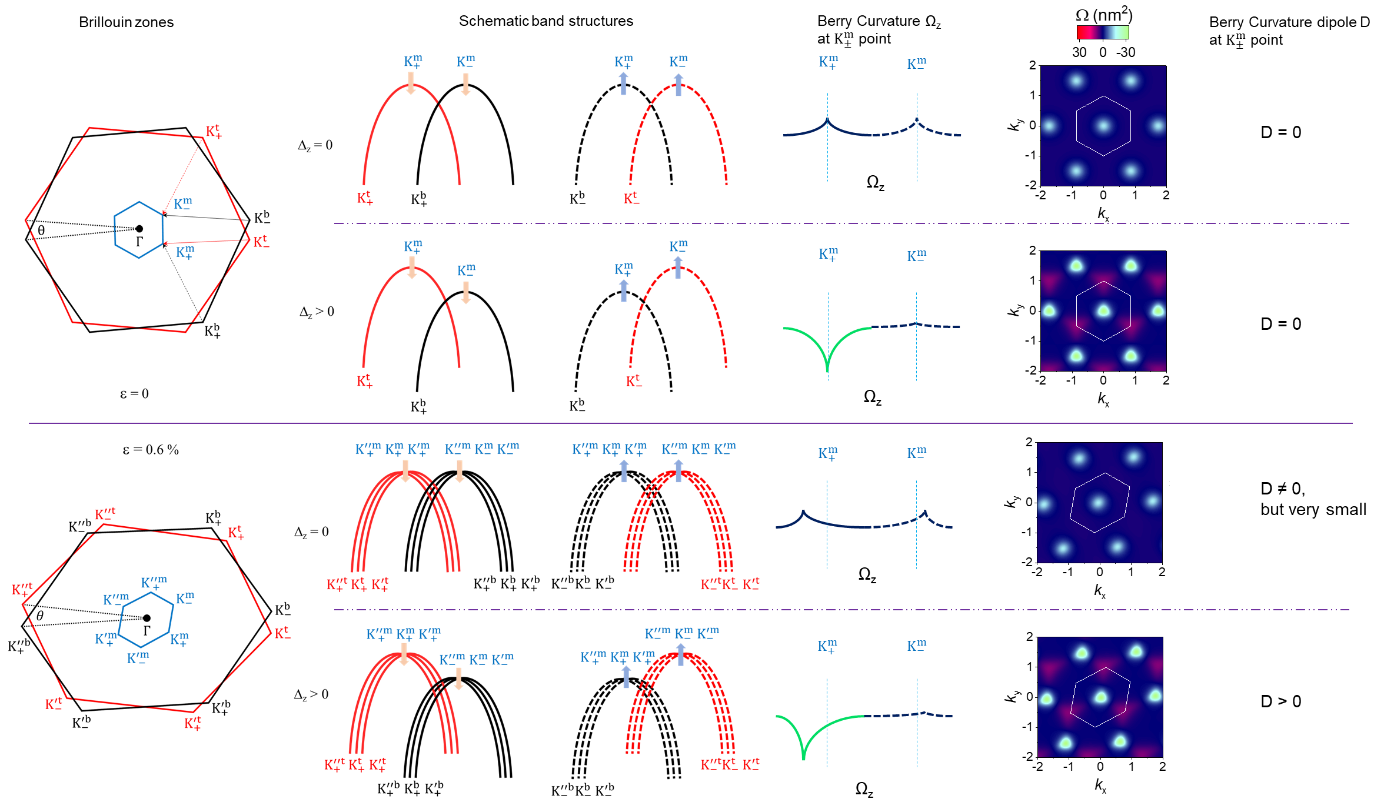
**

**Supplementary Fig. 6 | Strain and displacement electric field tunable Berry curvatures.**

1. є = 0, $\Delta_{z}$ = 0 meV : Ω_z_ is small, *D* = 0 (three-fold symmetry forces the dipole to vanish)
2. є = 0, $\Delta_{z}$ =30 meV : Ω_z_ is large, *D* = 0 (three-fold symmetry forces the dipole to vanish)
3. є = 0.6 %, $\Delta_{z}$ = 0 meV : Ω_z_ is small, *D* ≠ 0 (the BC hotspot is small, so dipole is small)
4. є = 0.6 %, $\Delta_{z}$ = 30 meV : Ω_z_ is large, *D* > 0 (the BC hotspot is large, so dipole is large)

**Supplementary Fig. 7 |** $\boldsymbol{\Delta R}$ **(top) and nonlinear Hall generation efficiency along different measurement directions (middle and bottom) versus filling.** To better illustrate the correlated insulating states, we plot the change of the resistance $\Delta R$ which is calculated by subtracting a background semiconductor resistance. The regime with $\Delta R>0$ is the insulating regime.


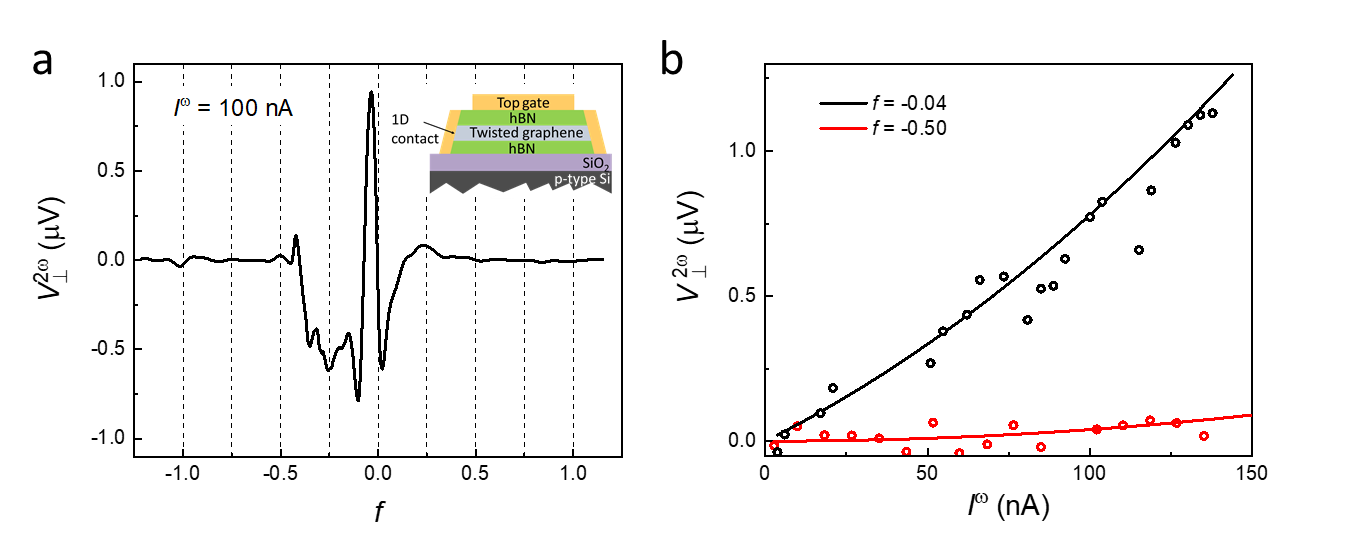


**Supplementary Fig. 8 | NHE in a 1.3° twisted bilayer graphene. a,** Filling dependent $V_{\perp}^{2\omega}$ measured at *T* =1.5 K. Inset: Schematic of the device structure. The nonlinear Hall generation efficiency $\eta=31 V^{-1}$ at $f=-0.04$ where $V_{\perp}^{2\omega}$ has its maximum value. **b,** $V_{\perp}^{2\omega}$ as a function of $I^{\omega}$ measured at different fillings. The dots are experimental data and the solid lines are parabolic fits of the data.

Though strain-induced nonlinear Hall signals can be detected in twisted bilayer graphene, the nonlinear Hall signal does not exhibit any giant behaviour near the half-filling. This is consistent with a theoretical prediction, saying that in twisted bilayer graphene, the nonlinear Hall signal becomes apparent only for twist angles very close to the first magic angle ∼1.1° (Ref. 6). Differently, there is no magic angle in twisted transition metal dichalcogenides: The bandwidth and BC hotspot can be tuned continuously along with the twist angle, demonstrating tWSe_2_ to be a more accessible system for introducing and manipulating nonlinear Hall signals.


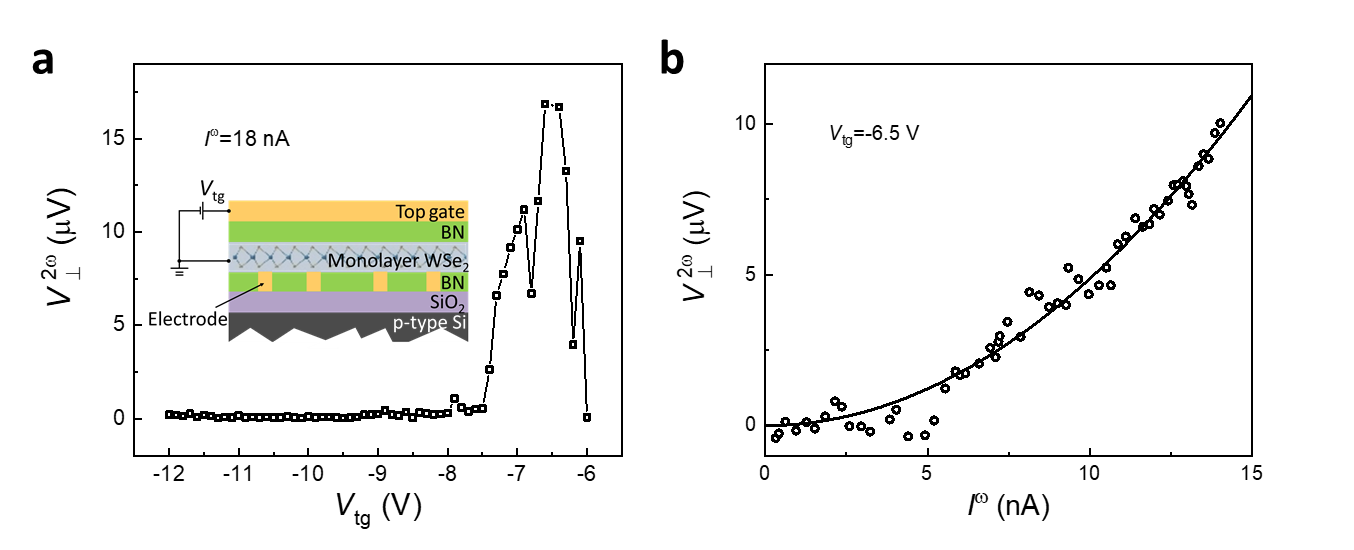


**Supplementary Fig. 9 | NHE in a monolayer WSe_2_. a,** Gate dependent $V_{\perp}^{2\omega}$ measured at *T* =1.5 K, where $V_{\mathrm{tg}}=-6V$ is the threshold voltage at which the Fermi level touches the band edge. Inset: Schematic of the device structure. **b,** $V_{\perp}^{2\omega}$ as a function of $I^{\omega}$ measured at $V_{\mathrm{tg}}=-6.5 V$. The dots are experimental data and the solid lines are parabolic fits of the data. The $V_{\perp}^{2\omega}$ signal in monolayer WSe_2_, although weak, indicates the universal strain-induced three-fold symmetry breaking in the fabricated devices.

**Supplementary Fig. 10 | Hall measurement in tWSe_2_.** Measured *R* (blue line in the upper panel), $\boldsymbol{\Delta R}$ **(red dashed line in the upper panel)** and Hall carrier density $n_{H}=-\left( 1/e \right)\left( {dR_{xy}}/{dB} \right)^{-1}$ as a function of gate voltage (or equivalently the filling *f* of the bands) at *T* = 1.5 K, where *R*_xy_ is the Hall resistance and *B* is the perpendicular magnetic field. $n_{H}$ changes sign at *V*_bg_ = 62 V and *V*_bg_ = 18.5 V, corresponding to the valence band edge *f* = 0 and the full-filling *f* = −1. Between them, three additional sign changes have been observed, where *f*_VHS_ labels the position of the single-particle band van Hove singularities (VHS), *f*_1_ and *f*_2_ labels the sign changes associated with correlated insulating states, indicating strong electron-electron correlations in tWSe_2_ system. Using the slope of the dashed line in the lower panel, we can get the gate induced carrier density at full-filling *p*_0_ ~ 2.61 × 10^12^ cm^-2^ and the twist angle $\theta=2.0$°.

**Supplementary Table 1 | NHE in different 2D systems.**

| Materials | Mechanism | $\eta= \frac{V_{\perp}^{2\omega}}{\left( V_{⫽}^{\omega} \right)^{2}}$  $(V^{-1})$ | $\frac{V_{\perp}^{2\omega}}{\left( I^{\omega} \right)^{2}}$  $(VA^{-2})$ | $\gamma=\frac{V_{\perp}^{2\omega}}{\left( I^{\omega} \right)^{2}R}=R\eta$  $(VW^{-1})$ | $\chi$ $=2\sigma\frac{L^{2}}{W}\frac{V_{\perp}^{2\omega}}{\left( V_{⫽}^{\omega} \right)^{2}}=2\sigma\frac{L^{2}}{W}\eta$  $\left( \mu mV^{-1}\Omega^{-1} \right)$ | Working Temperature (K) | Reference |
| --- | --- | --- | --- | --- | --- | --- | --- |
| tWSe_2_ | Nonlinear Hall effect | 1190 | $7.1\times{10}^{18}$ | $9.2\times{10}^{10}$ | $4.8\times{10}^{-4}$ | 1.5-40 | This work |
| Twisted bilayer graphene |  | 37 | $8\times{10}^{7}$ | $5.4\times{10}^{4}$ | 0.06 | 1.5-80 | This work Supplementary Fig. 8 |
| 1.26°Twisted graphene |  | 250 | $2\times{10}^{9}$ | $5\times{10}^{5}$ | 8.76 | 1.7-80 | Ref [7] |
| Graphene/BN superlattice |  | 110 | $3.9\times{10}^{7}$ | $2.2\times{10}^{4}$ | 1.1 | 1.5-120 | Ref [8] |
| Corrugated graphene |  | 6.25 | $1\times{10}^{8}$ | $2.5\times{10}^{4}$ | 0.03 | 3-15 | Ref [9] |
| Bilayer WTe_2_ |  | 2 | $2\times{10}^{8}$ | $2\times{10}^{4}$ | $9.4\times{10}^{-4}$ | 10-40 | Ref [2] |
| Few layer WTe_2_ |  | 0.005 | 7.2 | 0.19 | $3.0\times{10}^{-6}$ | 1.8-100 | Ref [10] |
| Monolayer WSe_2_ |  | 0.017 | $5\times{10}^{10}$ | $2.9\times{10}^{4}$ | $1.0\times{10}^{-7}$ | 1.5-90 | This work Supplementary Fig. 9 |
| Strained monolayer WSe_2_ |  | 0.1 | $6.25\times{10}^{5}$ | 250 | $1.6\times{10}^{-4}$ | 50-140 | Ref [11] |
| Bi_2_Se_3_ |  | 0.0000023 | 9 | 0.005 | $2.1\times{10}^{-7}$ | 2-200 | Ref [12] |
| Graphene | FET-based multipliers | 0.0006 | - | - | - | 300 | Ref [13] |

**Supplementary Table 2 | Fitting parameters in different twisted transition metal dichalcogenides samples.**

| System | α ($\Omega K^{-2}$) | *β* ($\Omega K^{-1}$) | Reference |
| --- | --- | --- | --- |
| 2.0° tWSe_2_ | 0.1 ~ 1.2 | 10 ~ 100 | Our work |
| 3.1° tWSe_2_ | ~ 5.5 | ~ 205 | Our work |
| 4.2° tWSe_2_ | 1 ~ 10 | 50 ~ 110 | Ref [14] |
| 4.5° tWSe_2_ ^*^ | ~ 0.2 | ~ 33 | Ref [14] |
| MoTe_2_/WSe_2_  (equals to 3.8° tWSe_2_) | 1 ~ 10000 | ~ 4940 ^#^ | Ref [15] |

^*^: The value given in the table for this sample are calculated from their Extended Fig. 1b since the range of the parameters are not given.

^#^: This value is calculated from their Extended Fig. 8 since the range of the parameter is not given.

**Supplementary Note 1 | comparison between the generation efficiency, responsivity, and nonlinear conductivity.**

We note that different parameters are defined to evaluate the strength of the nonlinear Hall signal in several references. Here, we make a comparison between those parameters.

1. In the pioneering work of the nonlinear Hall effect in bilayer WTe_2_ (Ref. 2), they use the responsivity defined as the ratio between the nonlinear output voltage and the input power $\gamma=\frac{V_{\perp}^{2\omega}}{\left( I^{\omega} \right)^{2}R}=\frac{V_{\perp}^{2\omega}}{\left( V_{⫽}^{\omega} \right)^{2}}R=R\eta$. This definition is mostly used combined with the noise equivalent power to describe the performance of a special device in the application field. Either a giant resistance or a giant nonlinear Hall generation efficiency ($\eta= \frac{V_{\perp}^{2\omega}}{\left( V_{⫽}^{\omega} \right)^{2}}$) can give rise to a giant responsivity.

2. In two recent works about the disorder-induced nonlinear transport in graphene superlattices^7, 8^, the nonlinear susceptibility $\chi=2\sigma\frac{L^{2}}{W}\frac{V_{\perp}^{2\omega}}{\left( V_{⫽}^{\omega} \right)^{2}}\propto\sigma\eta$ is introduced to measure the strength of the nonlinear Hall effect. Because a conductivity term $\sigma$ appears in the formula, either a giant conductance $\sigma$ or a giant nonlinear Hall generation efficiency $\eta$ can give rise to a giant nonlinear susceptibility. Especially, given the semi-metal characteristic of graphene, graphene-based systems generally possess a much larger conductivity than those in semiconducting WSe_2_-based systems. As listed in Supplementary Table. 1, the directly measured signal ${V_{\perp}^{2\omega}}/{\left( V_{⫽}^{\omega} \right)^{2}}$ in graphene system (both ours and others), is much smaller than that in the tWSe_2_ system.

3. Here in our work, similar to the definition of the spin Hall angle which measures the conversion efficiency of the charge currents to pure spin currents, we use the nonlinear Hall generation efficiency ${V_{\perp}^{2\omega}}/{\left( V_{⫽}^{\omega} \right)^{2}=\frac{e}{2\hbar^{2}Wp}D{m^{*}}/{m_{e}}}$ to measure the nonlinear Hall strengths, excluding the possibility that the giant nonlinear Hall generation efficiency is induced by a large sample resistance or conductance.

**Supplementary Note 2 | The continuum model in tWSe_2_.**

The moiré bands are captured by the continuum Hamiltonian $H=\sum_{\xi} \int dr \psi_{\xi}^{+}\left( r \right) \hat{H}_{\xi}\left( r \right)\psi_{\xi}(r)$ with

$$\hat{H}_{\xi}\left( r \right)=\left( \begin{matrix} \hat{H}_{b,\xi}+U_{b}\left( r \right) & T_{\xi}\left( r \right) \\ T_{\xi}^{+}\left( r \right) & \hat{H}_{t,\xi}+U_{t}\left( r \right) \end{matrix} \right)$$

here the field operator $\psi_{\xi}^{+}=\left( \psi_{b,\xi}^{+},\psi_{t,\xi}^{+} \right)$. $\xi$ is the valley index. $\hat{H}_{b\left( t \right),\xi}$ denotes the layer and valley dependent Hamiltonian and is given by $\hat{H}_{l,\xi}=-\frac{\hbar^{2}}{2m^{*}}\left( \hat{k}-K_{\xi,l}^{m} \right)^{2}-\frac{l\Delta_{z}}{2}$ where $l=b,t$ labels the bottom(top) layer, $m^{*}$ is the bare effective mass of the valence band, $\Delta_{z}$ is the staggered layer potential generated by the vertical displacement field. $U_{l}\left( r \right), T(r)$ represent the intralayer and interlayer moiré potential respectively and are given by

$$U_{l}\left( r \right)=V\sum_{i=1,2,3} e^{i\left( g_{i}\cdot r+l\psi\right)}+h.c.$$

$$T_{\xi}\left( r \right)=w\left( 1+e^{-i\xi g_{2}\cdot r}+e^{-i\xi\left( g_{1}+g_{2} \right)\cdot r} \right)$$

where the moiré reciprocal lattice vectors $g_{i}=\frac{4\pi}{\sqrt{3}L_{M}}\left( cos\frac{2\left( i-1 \right)\pi}{3},sin\frac{2\left( i-1 \right)\pi}{3} \right).$ The model parameters are $\left( m^{*},w,V,\psi\right)=(0.44m_{e},18meV,5meV,{91}^{^{\circ}})$^16, 17^. The staggered layer potential in tWSe_2_ can be calculated roughly by $\Delta_{z}=\frac{{C_{bg}V_{\mathrm{bg}}-C}_{tg}V_{\mathrm{tg}}}{C_{Q}}$, where $C_{Q}=\frac{2e^{2}m^{*}}{\pi\hbar^{2}}\approx{10}^{-4} F\mathrm{cm}^{-2}$ is the quantum capacitance of monolayer WSe_2_.

The strained twisted WSe_2_ is modeled by introducing a strain along the $\varphi$ direction with a strength $\epsilon$ on the bottom layer. The strain tensor $\boldsymbol{\varepsilon}$ can be written as $\boldsymbol{\varepsilon}=\epsilon\left( \begin{matrix} \cos^{2} \varphi-\upsilon\sin^{2} \varphi& \left( 1+\delta\right)\cos\varphi\sin\varphi\\ \left( 1+\delta\right)\cos\varphi\sin\varphi& \sin^{2} \varphi-\upsilon\cos^{2} \varphi\end{matrix} \right)$, where $\delta=0.19$ is the Poisson ratio^18^ of WSe_2_. Under this strain, the Dirac point for monolayer TMDC is shifted to $D_{\xi}=\left( I-\varepsilon\right)K_{\xi}-\xi A$ with the effective gauge field $A=\frac{\sqrt{3}}{2a_{0}}\beta\left( \epsilon_{xx}-\epsilon_{yy} , -2\epsilon_{xy} \right)$. $\beta$ is adopted as 2.30 in our calculation according to previous first principle calculation for strained WSe_2_ (Ref. ^19^). Then the continuum Hamiltonian of this strained WSe_2_ can be obtained as $\hat{H}_{b,\xi}=-\frac{\hbar^{2}}{2m^{*}}\left( \hat{k}-D_{\xi}^{m} \right)^{2}-\frac{\Delta_{z}}{2}$.

The Berry curvature dipole can be evaluated by:

$$\begin{aligned} D=-\int dk_{x}dk_{y} \sum_{\alpha,\xi} v_{\alpha k,\xi}^{x\left( y \right)}\Omega_{\alpha k,\xi}\delta_{F}\left( E_{\alpha}-E_{F} \right) \end{aligned}$$

where $\alpha, \xi$ are band and valley index. $\Omega_{\alpha k,\xi}=i\langle\partial_{\boldsymbol{k}}u_{\alpha k,\xi}|\times|\partial_{\boldsymbol{k}}u_{\alpha k,\xi}\rangle$ is the BC, and $|u_{\alpha k,\xi}\rangle$ is the Bloch wavefunction obtained from the continuum model. $v_{\alpha k,\xi}^{x\left( y \right)}$ is the band velocity and $\delta_{F}(E_{\alpha}-E_{F})$ is a delta function centered at $E_{F}$. Detailed calculations of the BCD for strained tWSe_2_ can be found in reference 20.

**Supplementary Note 3 | Sample fabrication process.**

1. Exfoliation of BN.
2. Clean marked wafers by sonication in acetone (10 mins) and IPA (10 mins).
3. Exfoliate BN on clean wafers and search for suitable flakes (thickness: 15~30 nm).
4. Heat clean at 800 °C for 6 hours in Ar:H_2_ (1:1) atmosphere.
5. Fabrication of bottom electrodes.
6. Spin coat two-layer PMMA resist. First, 495PMMA A3, 3000rpm for 1 min, baked at 150 °C for 3 mins. Second, 950PMMA A3, 3000rpm for 1 min, baked at 150 °C for 3 mins.
7. Pattern the bottom electrodes by EBL with 10kV acceleration voltage and 30 μm aperture size.
8. Develop in IPA:DI water (4 °C, 3:1 by volume) for 300s.
9. Reactive ion etching (RIE) under a CHF_3_ + O_2_ plasma conditions for 6s.
10. Deposit Pt (10 nm). The deposition began when the pressure reached 1.7E-7 torr, and the pressure was kept < 1E-6 torr during the deposition.
11. Lift off in acetone (40 °C) for 10 mins, followed by sonication in another acetone bath for 5 mins. Leave wafers in a clean acetone bath for more than 5 hours after sonication.
12. Heat clean at 200 °C for 3 hours in Ar:H_2_ (1:1) atmosphere.
13. Preparation of WSe_2_.
14. Clean wafers by sonication in acetone (10 mins) and IPA (10 mins).
15. Exfoliate monolayer WSe_2_ on clean wafers in a glove box with Ar atmosphere (the concentration of O_2_ and H_2_O are < 0.1 ppm) and search for suitable flakes (area > 300 μm^2^, clean, without wrinkles).
16. Cut the WSe_2_ into two parts using an AFM tip.
17. Transfer top BN and tWSe_2_. (Performed in glove box, usually done in 2 hours after the bottom electrodes are heat cleaned)
18. Pick up top BN by a clean PDMS/PC stamp at 70 °C.
19. Pick up top WSe_2_ at 90 °C after heating the stamp up to 110 °C.
20. Manually rotate the sample stage by a twist angle of about 1°–4° and pick up the bottom WSe_2_.
21. Align and stack the top parts to the bottom electrodes prepared in step 2.
22. Sample cleaning
23. Dissolve PC with chloroform (10 mins), another chloroform (15 mins), and IPA (10 mins).
24. Heat clean at 300 °C for 5 hours in Ar:H_2_ (1:1) atmosphere.
25. Fabrication of top gate and outer bonding pads.
26. Spin coat two-layer PMMA resist. First, 495PMMA A3, 3000rpm for 1 min, baked at 150 °C for 3 mins. Second, 950PMMA A3, 3000rpm for 1 min, baked at 150 °C for 3 mins.
27. Pattern the top gate by EBL with 10kV acceleration voltage and 30 μm aperture size.
28. Pattern the outer boning pads and connections by EBL with 10kV acceleration voltage and 120 μm aperture size.
29. Develop in IPA:DI water (4 °C, 3:1 by volume) for 300s.
30. Deposit Cr (5 nm) and Au (60 nm). The deposition began when the pressure reached 1.7E-7 torr, and the pressure was kept < 1E-6 torr during the deposition.
31. Lift off in acetone (40 °C) for 20 mins, followed by another acetone bath for 10 mins, and then leave wafers in a clean acetone bath for more than 5 hours.

**References**

1. Kerelsky A, McGilly LJ, Kennes DM, Xian L, Yankowitz M, Chen S*, et al.* Maximized electron interactions at the magic angle in twisted bilayer graphene. *Nature* **572**, 95-100 (2019).

2. Ma Q, Xu SY, Shen H, MacNeill D, Fatemi V, Chang TR*, et al.* Observation of the nonlinear Hall effect under time-reversal-symmetric conditions. *Nature* **565**, 337-342 (2019).

3. Du ZZ, Wang CM, Li S, Lu HZ, Xie XC. Disorder-induced nonlinear Hall effect with time-reversal symmetry. *Nat. Commun.* **10**, 3047 (2019).

4. Du ZZ, Wang CM, Lu HZ, Xie XC. Band Signatures for Strong Nonlinear Hall Effect in Bilayer WTe_2_. *Phys. Rev. Lett.* **121**, 266601 (2018).

5. Kadowaki K, Woods SB. Universal relationship of the resistivity and specific heat in heavy-Fermion compounds. *Solid State Commun.* **58**, 507-509 (1986).

6. Zhang C-P, Xiao J, Zhou BT, Hu J-X, Xie Y-M, Yan B*, et al.* Giant nonlinear Hall effect in strained twisted bilayer graphene. Preprint at https://arxiv.org/abs/2010.08333 (2020).

7. Duan J, Jian Y, Gao Y, Peng H, Zhong J, Feng Q*, et al.* Giant second-order nonlinearity in twisted bilayer graphene. Preprint at https://arxiv.org/abs/2201.09274 (2022).

8. He P, Koon GKW, Isobe H, Tan JY, Hu J, Neto AHC*, et al.* Graphene moiré superlattices with giant quantum nonlinearity of chiral Bloch electrons. *Nat. Nanotechnol.* **17**, 378-383 (2022).

9. Ho S-C, Chang C-H, Hsieh Y-C, Lo S-T, Huang B, Vu T-H-Y*, et al.* Hall effects in artificially corrugated bilayer graphene without breaking time-reversal symmetry. *Nat. Electron.* **4**, 116-125 (2021).

10. Kang K, Li T, Sohn E, Shan J, Mak KF. Nonlinear anomalous Hall effect in few-layer WTe_2_. *Nat. Mater.* **18**, 324-328 (2019).

11. Qin M-S, Zhu P-F, Ye X-G, Xu W-Z, Song Z-H, Liang J*, et al.* Strain Tunable Berry Curvature Dipole, Orbital Magnetization and Nonlinear Hall Effect in WSe_2_ Monolayer. *Chin. Phys. Lett.* **38**, 017301 (2021).

12. He P, Koon GKW, Isobe H, Tan JY, Hu J, Neto AHC*, et al.* Graphene moiré superlattices with giant quantum nonlinearity of chiral Bloch electrons. *Nat. Nanotechnol.* **17**, 378-383 (2022).

13. Wang H, Nezich D, Kong J, Palacios T. Graphene Frequency Multipliers. *IEEE Electron Device Lett.* **30**, 547-549 (2009).

14. Ghiotto A, Shih E-M, Pereira GSSG, Rhodes DA, Kim B, Zang J*, et al.* Quantum criticality in twisted transition metal dichalcogenides. *Nature* **597**, 345-349 (2021).

15. Li T, Jiang S, Li L, Zhang Y, Kang K, Zhu J*, et al.* Continuous Mott transition in semiconductor moiré superlattices. *Nature* **597**, 350-354 (2021).

16. Wu F, Lovorn T, Tutuc E, Martin I, MacDonald AH. Topological Insulators in Twisted Transition Metal Dichalcogenide Homobilayers. *Phys. Rev. Lett.* **122**, 086402 (2019).

17. Pan H, Wu F, Das Sarma S. Band topology, Hubbard model, Heisenberg model, and Dzyaloshinskii-Moriya interaction in twisted bilayer WSe_2_. *Phys. Rev. Research* **2**, 033087 (2020).

18. Zhang R, Koutsos V, Cheung R. Elastic properties of suspended multilayer WSe_2_. *Appl. Phys. Lett.* **108**, 042104 (2016).

19. Fang S, Carr S, Cazalilla MA, Kaxiras E. Electronic structure theory of strained two-dimensional materials with hexagonal symmetry. *Phys. Rev. B* **98**, 075106 (2018).

20. Hu J-X, Zhang C-P, Xie Y-M, Law KT. Nonlinear Hall Effects in Strained Twisted Bilayer WSe_2_. Preprint at <https://arxiv.org/abs/2004.14140> (2020).
